# Drastic variation in mitochondrial genome organization between two congeneric species of bird lice (Philopteridae: *Ibidoecus*)

**DOI:** 10.1186/s12864-024-11005-7

**Published:** 2024-11-14

**Authors:** Mei-Ling Cao, Yu Nie, Xi-Long Yi, Jun Xiong, Wei Wang, Yuan-Ping Deng, Yi-Tian Fu, Guo-Hua Liu, Renfu Shao

**Affiliations:** 1 Research Center for Parasites & Vectors, College of Veterinary Medicine, Hunan Agricultural University, Changsha, 410128 China; 2 College of Biotechnology, Hunan University of Environment and Biology, Hengyang, 421001 China; 3 Centre for Bioinnovation and School of Science, Technology and Engineering, University of the Sunshine Coast, Maroochydore, QLD 4556 Australia; 4 Department of Parasitology, Xiangya School of Basic Medicine, Central South University, Changsha, 410013 China

**Keywords:** Bird lice, Ischnocera, Mt genome fragmentation

## Abstract

The over 4,100 species of bird lice are classified into 214 genera in the parvorders Amblycera and Ischnocera. Congeneric species of bird lice usually share much similarity in morphology and in mitochondrial (mt) genome organization. Two recent studies, however, reported substantial intra-genus variation in mt genome organization in bird lice. Both the ancestral single-chromosome mt genome and a fragmented mt genome with two or three minichromosomes were observed in the genera *Austromenopon* and *Laemobothrion*. To better understand intra-genus variation in mt genome organization, we sequenced the complete mt genome of the white spoonbill louse *Ibidoecus plataleae* and compared it with that of the glossy ibis feather louse *Ibidoecus bisignatus* reported previously. We found that *I. plataleae* had a fragmented mt genome with 12 minichromosomes; each minichromosome was 2,798 to 3,628 bp in size and had 2 to 6 genes. This is in stark contrast to the mt genome of *I. bisignatus*, which has all genes on a single chromosome, 14,909 bp in size. This is the most drastic intra-genus variation in mt genome organization observed to date in animals, indicating an unprecedented rapid process of mt genome fragmentation in the genus *Ibidoecus*. The divergence time between *I. plataleae* and *I. bisignatus* is currently unknown but is estimated to be less than 23 million years. Either many minichromosal split events occurred after *I. plataleae* diverged from *I. bisignatus*, or one minichromosome splits into multiple minichromosomes in a single event. Sequencing and comparing more *Ibidoecusi* species will help understand the unusual mt genome fragmentation in this genus.

**Supplementary Information:**

The online version contains supplementary material available at 10.1186/s12864-024-11005-7.

## Introduction

The circular single-chromosome mitochondrial (mt) genome organization is highly conserved across eukaryotes [1, 2]. Nevertheless, multi-chromosome mt genome organization has been observed in Mesozoa [3], Nematoda [4, 5], Cnidaria [6, 7], Ichthyosporea [8], Euglenozoa [9], Porifera [10], and Insecta [11–13]. These multi-chromosome mt genomes comprise two to numerous minichromosomes, which are either circular or linear [14, 15]. For those eukaryotes that have multi-chromosome mt genome organization, i.e. fragmented mt genomes, species in the same genus usually all share this genomic feature except for several bird lice in the genera *Austromenopon* and *Laemobothrion*. Two recent studies showed that species of bird lice in these genera varied in mt genome organization. Dong et al. (2023) reported that *Austromenopon* sp. 1 (out of (ex) sooty shearwater) retained the single-chromosome mt genome organization while *Austromenopon* sp. 2 (ex sooty tern and crested tern) had a fragmented mt genome with two minichromosomes [16]. Sweet et al. (2021) reported that the mt genome of *Laemobothrion tinnunculi* (ex Australian hobby) comprised three minichromosomes [17]. Dong et al. (2023) [16], however, showed that three other *Laemobothrion* spp. (ex Eurasian coot, black kite, and Australian swamphen, respectively) all retained the single-chromosome mt genome organization.

There are over 4,100 species of bird lice classified into 214 genera of two parvorders: Amblycera and Ischnocera, parasitizing ~ 4,000 species of birds [18–30]. To date, 28 species in 18 genera of Amblycera [16, 17] and 40 species in 29 genera of Ischnocera [31–33] have been studied for mt genome organization. Fragmented mt genomes have been found in 20 species in 16 genera of Ischnocera and 11 species in seven genera of Amblycera; while the single-chromosome mt genome organization has been found in 17 species in 13 genera of Amblycera and 20 species in 14 genera of Ischnocera [16, 17, 31–33]. To better understand intra-genus variation in mt genome organization, we sequenced the complete mt genome of the white spoonbill louse *Ibidoecus plataleae*, and compared it with that of the glossy ibis head louse *Ibidoecus bisignatus* reported previously [33]. We report here the most drastic intra-genus variation in mt genome organization observed to date in animals: a fragmented mt genome with 12 minichromosomes in *I. plataleae* in contrast to a single-chromosome mt genome in *I. bisignatus*.

## Results

### Louse specimens collected from a white spoonbill were identified as *Ibidoecus plataleae*

Four species of lice in two families have been recorded parasitizing the white spoonbill, *Platalea leucorodia* Linnaeus, 1758: *Ardeicola plataleae* Linnaeus, 1758 (Philopteridae), *Ibidoecus plataleae* Denny, 1842 (Philopteridae), *Eucolpocephalum femorale* Piaget, 1880 (Menoponidae), and *Colpocephalum plataleae* Price and Beer, 1965 (Menoponidae) [18]. We identified the louse specimens we collected from a white spoonbill and used in the current study as *Ibidoecus plataleae* Denny, 1842. The host information in combination with the presence of filiform antenna and a short and stout body allowed us to identify tentatively the specimens to be *Ibidoecus plataleae* [18, 34]. This species identity was supported further by our observation of the following morphological characters in the specimens: (1) average body size of 2.30 mm for males and 3.10 mm for females; (2) average head size of 0.64 mm by 0.84 mm for males and 0.71 mm by 0.95 mm for females; (3) short preantennal region and long front margin of the head; (4) clypeal signature not aligned with the presence of a row of pustulated hairs across the entire posterior margin of the pterothorax; (5) pointed tergites; (6) distinctive round segment IX in males; (7) inner margin of the sternite on segment VII connected to the genital plate of females; and (8) the shape and thickening of the sclerites that support the vulva laterally [34–38].

### The mitochondrial genome of *Ibidoecus plataleae* is fragmented and comprises 12 circular minichromosomes

**Fig. 1:**
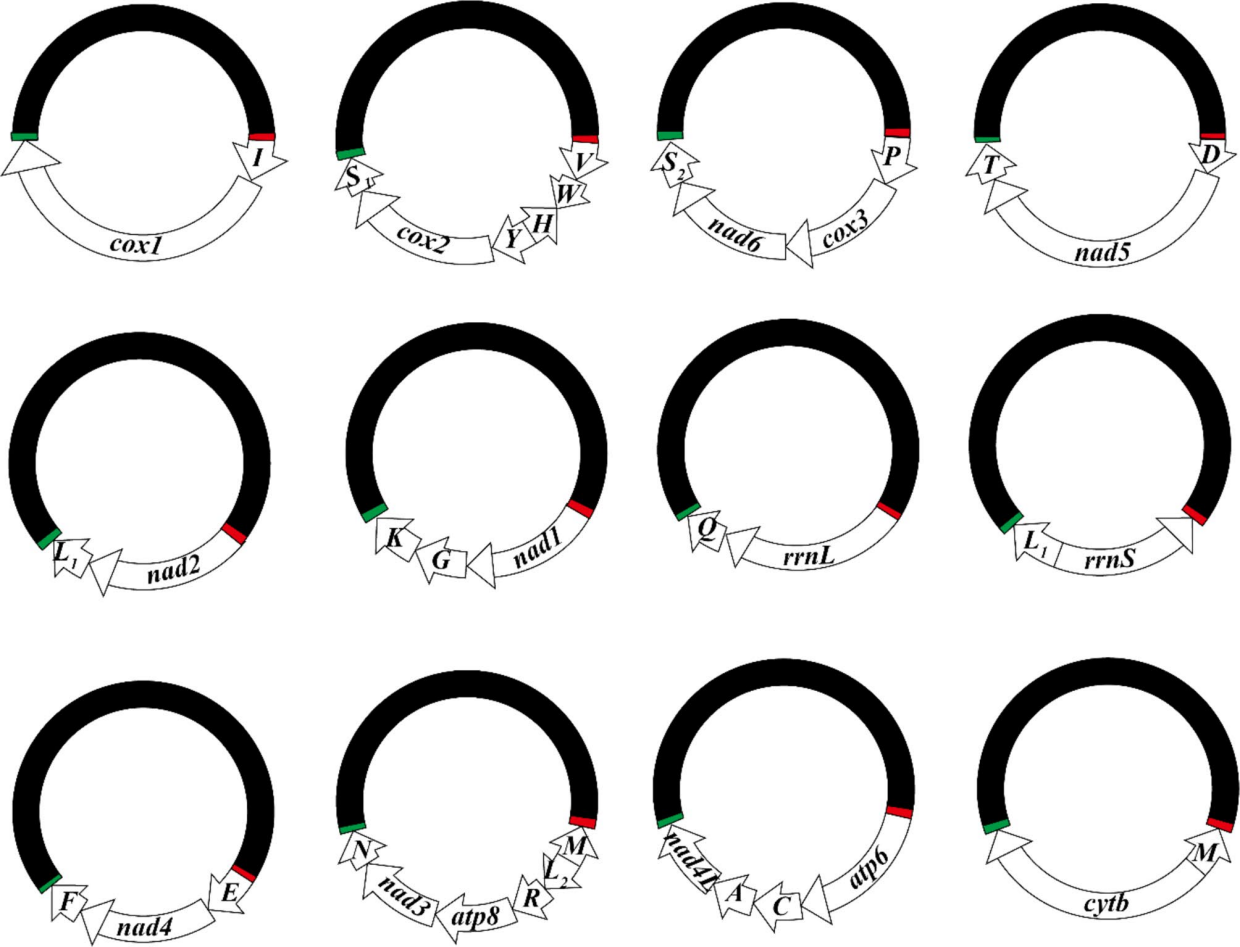
The complete mitochondrial genome of the white spoonbill louse, *Ibidoecus plataleae*. Each minichromosome has a coding region and a non-coding region (NCR, in black). The red and green bars represent the upstream and downstrea of NCR respectively

14,932,876 pairs of raw sequence reads were generated with Illumina HiSeq 2500 platform; after filtering, 13,529,268 pairs of clean reads were obtained. Assembly of the clean reads produced the complete mt genome of *I*. *plataleae*: all of the 37 mt genes typically found in animals were identified including 13 PCGs, 2 rRNA genes and 22 tRNA genes (two of them, *trnL*_*1*_ and *trnM*, were identically duplicated). These mt genes were located on 12 minichromosomes; each minichromosome was 2.8–3.6 kb in size, was circular and had 2–6 genes and a large non-coding region (NCR) (Table 1; Fig. 1). The gene-containing region ranges from 782 to 1,598 bp in size, while the NCR ranges from 1,670 to 2,113 bp (Table 1; Fig. 1). All genes are transcribed in the same direction relative to the NCR except for *trnM*, *trnH* and *rrnS*, which are transcribed in the opposite direction (Fig. 1). Each of the 12 circular minichromosomes was verified by PCR using specific outbound primers designed from each minichromosome. The amplicons from the 12 mt minichromosomes were sequenced individually using HiSeq 2500 platform; 13,529,268 pairs of clean sequence reads were generated and assembled to produce the full-length nucleotide sequence of each minichromosome. The mt minichromosomal sequences of *I*. *plataleae* were annotated and deposited in GenBank under accession numbers ON585561-72.

**Table 1:** The mitochondrial minichromosomes of the white spoonbill louse, *Ibidoecus plataleae*

Minichromosome	Size (bp)	Size of the region with genes (bp)	Size of the large non-coding region (bp)	Size of intergenic regions (bp)
*rrnS* *-L*_*1*_	2798	782	2016	0
*rrnL-Q*	3359	1246	2111	2
*nad1*-*G-K*	3187	1074	2113	0
*nad2-L*_*1*_	3101	1010	2026	64
*M* *-L*_*2*_ *-R-atp8-nad3-N*	2847	839	1728	280
*E-nad4-F*	3436	1423	2002	11
*D-nad5-T*	3628	1759	1845	24
*P-cox3-nad6-S*_*2*_	3505	1374	2045	86
*V-W-* *H* *-Y-cox2-S*_*1*_	3078	1009	2042	27
*I-cox1*	3554	1598	1956	0
*M* *-cytb*	3176	1161	1941	74
*atp6-C-A-nad4L*	2955	1125	1670	160
Total	38,623	14,400	23,495	728

*Note*: genes underlined are transcribed in opposite orientation to those not underlined

### *Ibidoecus plataleae* and *Ibidoecus bisignatus* differ drastically in mitochondrial genome organization but share six derived gene clusters

**Fig. 2:**
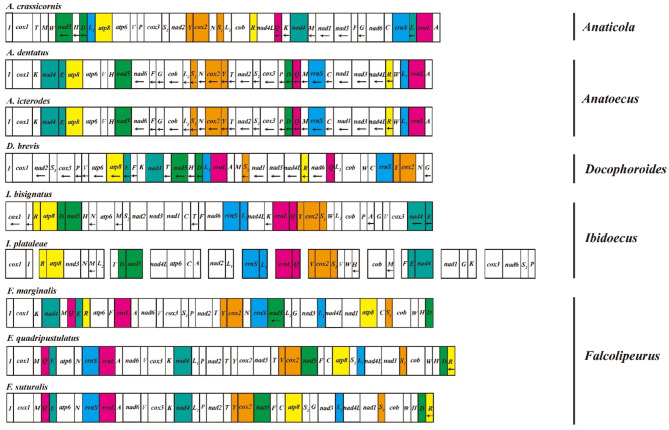
Linearized mitochondrial genome of Ischnocera lice. In order to better identify the gene blocks shared by *Ibidoecus* genus, we used different color markers. And the same color markers were used in other non-*Ibidoecus* genus species, in order to find out whether the sharing of *Ibidoecus* genus gene block is also found in other species

**Fig. 3:**
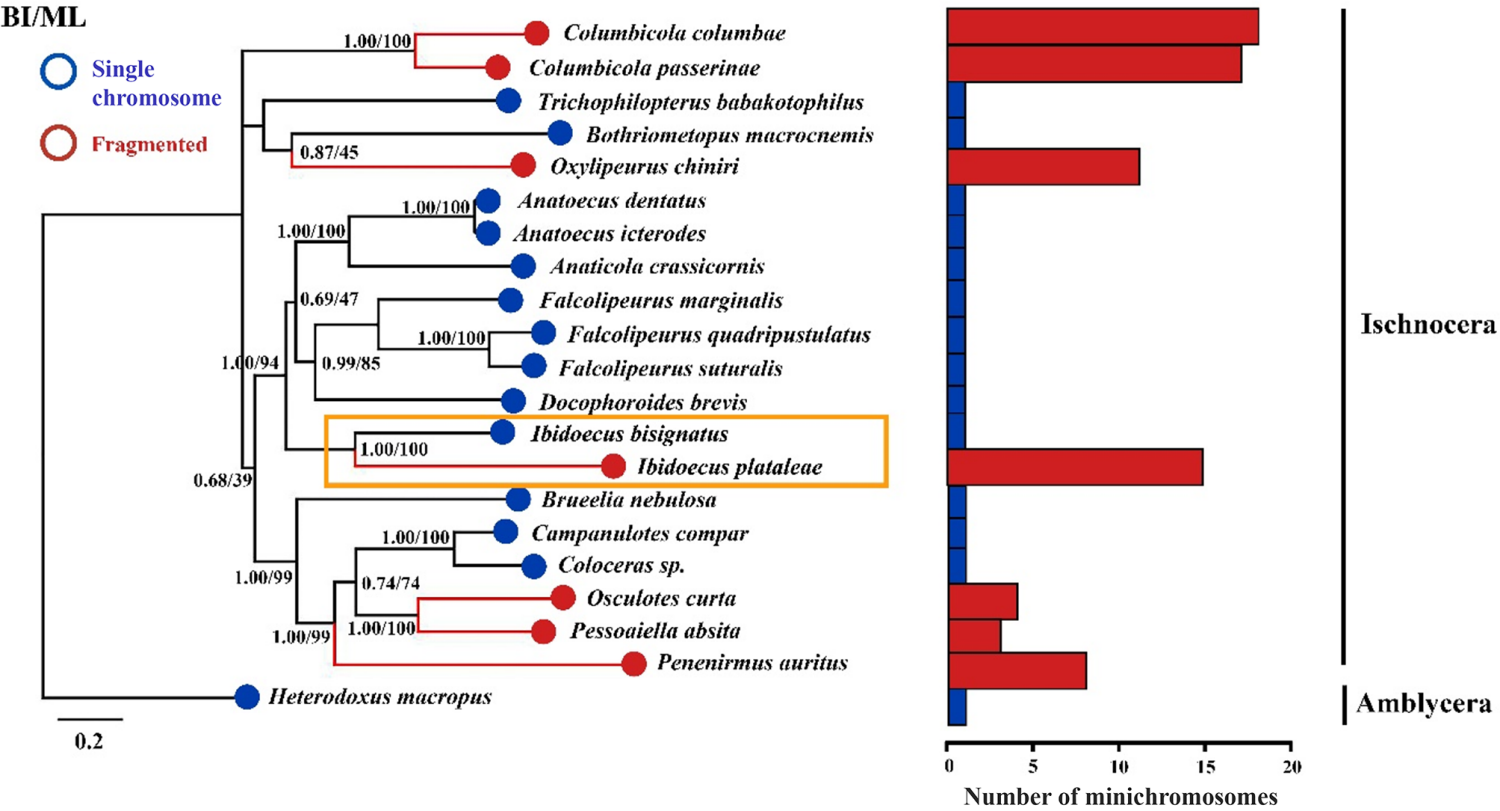
Phylogenetic relationships among 21 species of lice inferred by Bayesian inference and Maximum likelihood from concatenated amino acid sequences of 13 protein-coding genes. Wallaby louse, *Heterodoxus macropus* was as an outgroup. Bayesian posterior probabilities (Bpp) and bootstrapping frequencies (Bf) were indicated at nodes. The bar at the bottom left means distance scale. And red and blue circles represent fragmented and unfragmented mitochondrial genomes respectively. The bars on the right means the number of minichromosomes with a quantity scale on the bottom

The mt genome organization of *Ibidoecus plataleae* differs drastically from that of the glossy ibis head louse, *Ibidoecus bisignatus*. The 37 mt genes of *I. bisignatus* are on a single circular chromosome, 14,909 bp in size [33], which is typical of animals [1, 11]. These two *Ibidoecus* species also differ substantially in their mt gene sequences with their protein-coding and rRNA gene sequences differing by 30.40–53.73% (Table 2). Despite these differences, *I. bisignatus* and *I. plataleae* share six derived gene clusters with each other: *R-atp8*, *D-nad5*, *rrnS-L*_*1*_, *rrnL-Q*, *Y-cox2-S*_*1*_, and *E-nad4* (Fig. 2). These six gene clusters are not seen in any other parasitic lice reported to date and are potentially synapomorphies for *Ibidoecus* species. Our phylogenetic analyses also strongly support the close relationship between *I. bisignatus* and *I. plataleae* relative to bird lice in other genera of Ischnocera (Fig. 3). Bayesian inference (BI) and maximum likelihood (ML) trees are consistent in the tree topology. Species in the genera *Ibidoecus*, *Anatoecus*, *Anaticola*, *Falcolipeurus* and *Docophoroides* formed a well-supported clade, in which *I. plataleae* is the only species that has a fragmented mt genome with 12 minichromosomes while all other species retained the single-chromosome mt genome organization (Fig. 3). Outside this clade, fragmented mt genomes are found in bird lice in five other genera of Ischnocera (*Osculotes*, *Pessoaiella*, *Penenirmus*, *Oxvlipeurus*, *Columbicola*) whereas bird lice in the genera *Brueelia*, *Campanulotes*, *Coloceras*, *Trichophilopterus* and *Bothriometopus* also retained the ancestral single-chromosome mt genome organization (Fig. 3).

**Table 2:** Base differences in mitochondrial genes between *Ibidoecus plataleae* (*Ip*) and *Ibidoecus bisignatus* (*Ib*)

Gene	Gene size (bp)		Base differences between Ip and Ib (%)
	*Ip*	*Ib*	
*atp6*	720	696	47.78
*atp8*	174	183	53.01
*cox1*	1533	1524	30.40
*cox2*	681	675	35.98
*cox3*	789	783	39.29
*cytb*	1092	1089	41.85
*nad1*	941	894	41.98
*nad2*	942	948	48.04
*nad3*	402	339	53.73
*nad4*	1294	1302	46.93
*nad4L*	267	268	47.01
*nad5*	1629	1684	45.13
*nad6*	447	453	50.77
*rrnL*	1180	1130	34.92
*rrnS*	713	721	40.08

## Discussion

The over 4,100 species of bird lice listed in Price et al. (2003) [18] and subsequent studies were classified into 214 genera of five families in the parvorders Amblycera and Ischnocera; species in the same genus tended to share much similarity in morphology and mt genome organization [16–18]. The current study reports the first case of drastic variation in mt genome organization between two species of bird lice in the same genus. To our knowledge, this is the most drastic intra-genus variation in mt genome organization observed to date in animals. Only two other cases of intra-genus variation in mt genome organization have been reported previously in bird lice. Dong et al. (2023) reported that *Austromenopon* sp. 1 (ex sooty shearwater) retained the single-chromosome mt genome organization whereas *Austromenopon* sp. 2 (ex sooty tern and crested tern) had a fragmented mt genome with two minichromosomes [16]. Sweet et al. (2021) [17] reported that the mt genome of *Laemobothrion* (*Laemobothrion*) *tinnunculi* (ex Australian hobby) comprised three minichromosomes. Dong et al. (2023) [16], however, showed that three other *Laemobothrion* spp. (ex Eurasian coot, black kite, and Australian swamphen, respectively) all retained the single-chromosome mt genome organization. No intra-genus variation in mt genome organization has been observed in other genera of bird lice for which multiple species have been studied to date for mt genome organization. These genera include *Actornithophilus*, *Colpocephalum*, *Franciscoloa* and *Myrsidea*; the multiple species in each of these genera either all have fragmented mt genomes or all retain the single-chromosome mt genome organization [16, 17].

Fragmented mt genomes have also been observed in numerous species of mammal lice from different genera and in a few species of book lice in the genus *Liposcelis*. Without any exception, all eutherian mammal lice studied to date in 13 genera of the parvaorders Anoplura, Rhynchophthirina and Trichodectera have fragmented mt genomes [11, 16, 39–49]. *Heterodoxus* is the only genus of mammal lice to date in the parvorder Amblycera for which multiple species have been studied for mt genome organization: both *H. macropus* and *H. spiniger* retain the single-chromosome mt genome organization [17, 50]. Sweet et al. (2021) [17] reported fragmented mt genomes in two genera of mammal lice (*Cummingsia* and *Macrogyropus*) of the parvorder Amblycera; however, only a single species has been studied for mt genome organization to date for each of these genera. For book lice in the genus *Liposcelis*, six species have been studied to date for mt genome organization: while *L. decolor* and *L. pearmani* retain the single-chromosome mt genome organization, *L. bostrychophila*, *L. brunnea*, *L. entomophila* and *L. paeta* all have fragmented mt genomes with two or three minichromosomes [51].

It is evident that mt genome fragmentation occurred once in the most recent common ancestor (MRCA) of eutherian mammal lice in Anoplura, Rhynchophthirina and Trichodectera about 60 MYA [33, 46]. Many events of mt genome fragmentation, however, have occurred independently in mammal lice and bird lice in Amblycera and Ischnocera [16, 33]. Fragmentation of mt genome in the book lice in the genus *Liposcelis* is also an independent event from those in the parasitic lice of mammals and birds [33, 46]. The limited evidence available to date supports the hypothesis that mt genome fragmentation is a gradual process starting with the ancestral single-chromosome mt genome splitting into two minichromosomes, followed by further minichromosomal split events, and in some lineages, minichromosomal merger events [39]. Two observations made separately in bird lice and book lice both support directly the initial step of mt genome fragmentation: (1) *Austromenopon* sp. 1 (ex sooty shearwater) retains the ancestral single-chromosome mt genome organization whereas *Austromenopon* sp. 2 (ex sooty tern and crested tern) has a fragmented mt genome with two minichromosomes [16]; and (2) *Liposcelis decolor* and *L. pearmani* retain the ancestral single-chromosome mt genome organization, whereas *L. bostrychophila*, *L. entomophila* and *L. paeta* all have fragmented mt genomes with two minichromosomes [51]. After the initial step of mt genome fragmentation, minichromosomes may split further, producing more minichromosomes, or in some lineages, two minichromosomes merge as one, reducing the number of minichromosomes. Evidence for minichromosomal splitting has been observed in chimp lice, human lice, guanaco lice and rat lice; and evidence for minichromosomal merging has been observed in horse lice and pig lice [39]. A model of gene degeneration followed by deletion was proposed to account for how one minichromosome could split into two minichromosomes based on evidence observed in the human pubic lice [41]. On the timeframe and pace of mt genome fragmentation, Herd et al. (2015) [45] showed that a single minichromosome fragmented into three in about 6 million years in the lineage leading to the human lice after this lineage diverged from the lineage leading to the chimp lice.

The drastic variation in mt genome organization between *Ibidoecus plataleae* and *Ibidoecus bisignatus* observed in the current study indicates that mt genome fragmentation in *I. plataleae* is likely a very rapid process not yet seen previously in any other parasitic lice and the book lice. The exact divergence time between *I. plataleae* and *I. bisignatus* is unknown but is estimated to be less than 23 MY [33]. Either many minichromosal split events occurred after *I. plataleae* diverged from *I. bisignatus*, or one minichromosome splits into multiple minichromosomes in a single event. The genus *Ibidoecus* contains 23 species of lice that parasitize a range of ibis species, two spoonbill species and a limpkin species [18]. Sequencing and comparing the mt genomes of more *Ibidoecusi* species will help understand further the unusual mt genome fragmentation process in this genus.

## Materials and methods

### Sample collection and DNA extraction

A total of 60 individual lice were collected from a white spoonbill, *Platalea leucorodia* Linnaeus, 1758 in Beijing Wildlife Rescue Center, China. The louse specimens were identified as *Ibidoecus plataleae* Denny, 1842 based on host information and morphological features [43]. The louse specimens were imaged, washed with physiological saline solution, immersed in 95% (v/v) ethanol, and then stored in a -40 °C freezer. The individual lice were pooled together, from which genomic DNA was extracted using the DNeasy Tissue Kit (Promega, Madison, USA) according to the manufacturer’s instructions. A fragment of *cox*1 and a fragment of *rrn*S were amplified by PCR using conserved primers reported in Shao et al. (2017) [39] and were sequenced using the Sanger method. The sequences of these fragments were later used as the initial references for high-throughput sequence-read assembly.

### High-throughput sequencing, assembly and annotation

The genomic DNA extracted above was sequenced using Illumina HiSeq 2500 (insert size 300 bp, paired-end, read length 250 bp each) at Novogene Bioinformatics Technology Co., Ltd (Beijing, China). Genomic DNA samples are fragmented by sonication to a size of 300 bp. Then DNA fragments are end-repaired, A-tailed, and ligated with the full-length adapter for Illumina sequencing. Subsequently, further PCR amplification is performed. The PCR products are purified by the AMPure XP system (Beverly, USA). After the library quality is assessed on the Agilent 5400 system (Agilent, USA) and quantified by QPCR (1.5 nM), sequencing is performed on the Illumina platform with the PE250 strategy. The original fluorescence image files obtained from the Illumina platform are converted to short reads (raw data) by base calling. These short reads are recorded in FASTQ format. The sequences of *cox*1 and *rrn*S fragments obtained above were used as initial references for assembly (mapping assembly) of these two genes and their upstream and downstream genes with Geneious 11.1.5 [52]. The assembly parameters were set at a minimum overlap identity of 99% and a minimum overlap of 150 bp. NCBI Open Reading Frame (ORF) Finder (https://www.ncbi.nlm.nih.gov/orffinder/) was used to identify potential protein-coding genes (PCG). Basic Local Alignment Search Tool (BLAST: https://blast.ncbi.nlm.nih.gov/Blast.cgi) was used to determine PCGs and rRNAs. tRNAs were identified using ARWEN [53] and tRNAscan-SE [54]. Besides all the genes were also verified by MITOS 2 (MITOS Web Server (uni-leipzig.de)).

### Verification of mitochondrial minichromosomes and sequence analysis

Mitochondrial minichromosomes identified by sequence analysis were validated by PCR with specific primers designed from each minichromosome. A pair of outbound primers (a forward primer and a reverse primer) were designed to amplify a minichromosome in full length or near full length if the minichromosome is in a circular organization. PCR conditions were as follows: 94 °C pre-denaturation for 1 min; then 98 °C denaturation for 10 s, annealing at 55 ~ 68 °C for 40 s, extension at 68 °C for 4 min, repeating for 25 to 35 cycles; and a final extension at 72 °C for 8 min before storage at 4 °C. The size of PCR amplicons was checked by agarose gel (1%) electrophoresis. Amplicons from each minichromosome were sequenced individually using Illumina HiSeq 2500 at Novogene Bioinformatics Technology Co., Ltd (Beijing, China) and assembled as detailed above for genomic DNA. The minichromosome structure and the linearized mitochondrial genome are both drawn with Adobe Photoshop 6.0 [55]. And the base differences of sequence are calculated with MEGA X [56].

### Phylogenetic analysis

Amino acid sequences of 13 mt PCGs of 21 species of parasitic lice were aligned using MAFFT (https://mafft.cbrc.jp/alignment/server/). Only about 1700 aa of each lice species were retained after poorly aligned regions (about 1900 aa) were trimmed with Gblocks with a setting that ‘For a more stringent selection: Do not allow many contiguous nonconserved positions’ (https://www.phylogeny.fr/one_task.cgitask_type=gblocks). The best fit models were selected using ProtTest 3.4 [57]; maximum likelihood tree (ML) and Bayesian tree (BI) were constructed using PhyML v 3.0 [58] and MrBayes v 3.2.6 [59], respectively. 100 bootstrap replicates were computed in ML analysis. For BI analysis, 1,000,000 generations were run, with a tree being generated every 100 generations, and generations of 2500 was used for burn-in and calculation. Phylogenetic trees were drawn using FigTree v.1.41 (http://tree.bio.ed.ac.uk/software/figtree/); the wallaby louse, *Heterodoxus macropus*, was used as the outgroup to root the trees.

## Electronic supplementary material

Below is the link to the electronic supplementary material.

Supplementary Material 1

## Data Availability

The sequence data generated in this study have been deposited in GenBank (accession numbers ON585561 -72).
