# Supplementary material for: Drastic variation in mitochondrial genome organization between two congeneric species of bird lice (Philopteridae: Ibidoecus)
Source: BMC Genomics. 2024 Nov 14;25:1084. doi: 10.1186/s12864-024-11005-7 (PMC11566740; doi:10.1186/s12864-024-11005-7)
Supplement: Supplementary file 1 — Supplementary Material 1 [file 12864_2024_11005_MOESM1_ESM.docx]

**Supporting Information:**

Additional supporting information may be found online in the Supporting Information section at the end of the article.

**Supplementary Table 1**: Primers used in PCR validation of the mitochondrial minichromosomes of the white spoonbill louse, *Ibidoecus plataleae*.

**Supplementary Table 2**: Species of parasitic lice included in the phylogenetic analysis in this study.

**Supplementary Table 3**: Species of bird lice described after 2003.

**Supplementary Fig. 1**: The dorsal and ventral sides of a female louse of Ibidoecus plataleae.

**Supplementary Fig. 2**: Secondary structures of the tRNAs.

**Supplementary Table 1** Primers used in PCR validation of the mitochondrial minichromosomes of the white spoonbill louse, *Ibidoecus plataleae*

| Primer | Sequence (5′–3′) | Size of amplified region (bp) |
| --- | --- | --- |
| SF | AGCGGTAACATTTCGTCTTTGCT | ⁓3000 |
| SR | GTTTAAGTAAGGTTGTAGCGTAG |  |
| LF | GTCTGGAATAAATGGTTTGACGAGGTT | ⁓3000 |
| LR | ATAAAGCACTGAGCAGGCACGAC |  |
| N1F | GTGGGCAGGGTATCTTATGTATT | ⁓3000 |
| N1R | CAGACCAGCATAACGGGAAAGTA |  |
| N2F | TTCGAGTTGAAAGTATTACGTGGTT | ⁓3000 |
| N2R | GATTCCTACTAAACTGAGGGATAAA |  |
| N3F | TTCCAAAACATATTCGCTCCTAC | ⁓3000 |
| N3R | AAACCAGAAACAGAATCCCCAAA |  |
| N4F | GCCTTGAGTATAGTGTTGACCTT | ⁓3000 |
| N4R | TTACTGTCTTTACTCGCTCACCC |  |
| N5F | AGCGGCTAGAATAACTAAGAGGG | ⁓3000 |
| N5R | GACCACCAAGCATAACGAGCAAA |  |
| C3F | TGATGTGGAACTCGGATGTATGT | ⁓3000 |
| C3R | TTACATCACGCCACCACAAAGAA |  |
| C2F | AATGTTTCGATTACTTGAGGTCG | ⁓3000 |
| C2R | AGCATGTACGAGCTAAACTCAAC |  |
| C1F | TTTTATCACCAGCCCTTCTTTGA | ⁓3000 |
| C1R | CTCTGGTAAAGTAAGCCCGTCTA |  |
| CYF | GGACAACTTTATGGAGGCGAACC | ⁓3000 |
| CFR | CGGATCTATCAGCACATCGTGGC |  |
| A6F | GGTTCTTTATCCGCCCTATTTCA | ⁓3000 |
| A6R | AAATGGTGCTAGTACCAGAGGTG |  |

**Supplementary Table 2** Species of parasitic lice included in the phylogenetic analysis in this study

| Species | GenBank # | PMID/Author | Year |
| --- | --- | --- | --- |
| *Heterodoxus macropus* | AF270939 | 11319269 | 2001 |
| *Anaticola crassicornis* | ON584173 | Nie Y | 2022 |
| *Anatoecus dentatus* | NC_069918 | Nie Y | 2022 |
| *Anatoecus icterodes* | NC_065453 | [35804150](https://www.ncbi.nlm.nih.gov/pubmed/35804150) | 2022 |
| *Brueelia nebulosa* | NC_071830 | Sweet AD | 2022 |
| *Docophoroides brevis* | NC_065456 | [35804150](https://www.ncbi.nlm.nih.gov/pubmed/35804150) | 2022 |
| *Falcolipeurus marginalis* | NC_065457 | 35804150 | 2022 |
| *Falcolipeurus quadripustulatus* | MH001226 | 30239978 | 2018 |
| *Falcolipeurus suturalis* | MW696813 | [34016171](https://www.ncbi.nlm.nih.gov/pubmed/34016171) | 2021 |
| *Ibidoecus bisignatus* | NC_015999 | [21813020](https://www.ncbi.nlm.nih.gov/pubmed/21813020) | 2011 |
| *Ibidoecus plataleae* | ON585561~72 | Present study | 2022 |
| *Osculotes curta* | ON643933~36 | [35804150](https://www.ncbi.nlm.nih.gov/pubmed/35804150) | 2022 |
| *Oxylipeurus chiniri* | ON643937~46 | [35804150](https://www.ncbi.nlm.nih.gov/pubmed/35804150) | 2022 |
| *Penenirmus auritus* | ON643953~60 | [35804150](https://www.ncbi.nlm.nih.gov/pubmed/35804150) | 2022 |
| *Pessoaiella absita* | ON643991~93 | 35804150 | 2022 |
| *Trichophilopterus babakotophilus* | ON643990 | 35804150 | 2022 |
| *Campanulotes compar* | NC_056779 | 30239978 | 2018 |
| *Coloceras sp.* | JN122000 | 21813020 | 2011 |
| *Bothriometopus macrocnemis* | EU183542 | 17925995 | 2007 |
| *Columbicola columbae* | MT113910~25 | 32231878 | 2020 |
| *Columbicola passerinae* | MT094283~99 | 32231878 | 2020 |

**Supplementary Table 3** The number of species of bird lice after 2003

| number | Reference | time |
| --- | --- | --- |
| 1 | Adly E, Nasser M, Soliman D, Gustafsson DR, Shehata M. New records of chewing lice (Phthiraptera: Amblycera, Ischnocera) from Egyptian pigeons and doves (Columbiformes), with description of one new species. Acta Trop. 2019 Feb;190:22-27. doi: 10.1016/j.actatropica.2018.10.016 | 2018 |
| 1 | Alahmed A, Aldryhim Y, Shobrak M, Nasser M. A new species of the genus *Amyrsidea* (Phthiraptera: Amblycera: Menoponidae) parasitizing domestic chickens in Saudi Arabia. Zootaxa. 2017 Mar 2;4238(2):zootaxa.4238.2.5. doi: 10.11646/zootaxa.4238.2.5 | 2017 |
| 1 | Bansal N, Ahmad A, Arya G, Khan V, Saxena AK. Menacanthus palmai, a new species of chewing louse (Menoponidae: Amblycera: Phthiraptera) from the Coturnix coromandelica. J Parasit Dis. 2013 Oct;37(2):276-80. doi: 10.1007/s12639-012-0179-z | 2013 |
| 8 | Bush SE, Price RD, Clayton DH. Descriptions of eight new species of feather lice in the genus Columbicola (Phthiraptera: Philopteridae), with a comprehensive world checklist. J Parasitol. 2009 Apr;95(2):286-94. doi: 10.1645/GE-1799.1 | 2009 |
| 2 | Bush SE, Price RD. Reconsideration of the longiceps species group of the feather louse genus Columbicola (Phthiraptera: Philopteridae) with descriptions of two new species. J Parasitol. 2006 Oct;92(5):949-52. doi: 10.1645/GE-832R.1 | 2006 |
| 1 | Cicchino AC, González-Acuña D. A new species of Brueelia Kéler (Phthiraptera: Philopteridae) parasitic on the Common Diuca-Finch, Diuca diuca diuca (Aves: Emberizidae) in Chile. Neotrop Entomol. 2009 Jul-Aug;38(4):504-7. doi: 10.1590/s1519-566x2009000400011 | 2009 |
| 2 | Cicchino AC, González-Acuña D. Two new species of Brueelia (Phthiraptera: Philopteridas s. l.) parasitic on two species of Phrygilus (Aves: Emberizidae) from Chile. Neotrop Entomol. 2008 May-Jun;37(3):301-4. doi: 10.1590/s1519-566x2008000300009 | 2008 |
| 1 | Cicchino AC, González-Acuña DA. A new species of Aquanirmus Clay & Meinertzhagen (Phthiraptera: Philopteridae) parasitic on the Great Grebe, Podiceps major (Aves: Podicipedidae), in Argentina and Chile. Neotrop Entomol. 2009 May-Jun;38(3):384-8. doi: 10.1590/s1519-566x2009000300014 | 2009 |
| 1 | Cicchino AC, Valim MP, González-Acuña D. A review of the louse genus Tinamotaecola (Insecta: Phthiraptera: Philopteridae sensu lato), with description of a new species. Zootaxa. 2014 Jul 11;3835(3):349-63. doi: 10.11646/zootaxa.3835.3.4 | 2014 |
| 1 | Deng YP, Wang W, Fu YT, Nie Y, Xie Y, Liu GH. Morphological and molecular evidence reveals a new species of chewing louse *Pancola ailurus* n. sp. (Phthiraptera: Trichodectidae) from the endangered Chinese red panda *Ailurus styani*. Int J Parasitol Parasites Wildl. 2022 Dec 27;20:31-38. doi: 10.1016/j.ijppaw.2022.12.004 | 2022 |
| 3 | Gustafsson DR, Adam C, Zou F. Erratum: DANIEL R. GUSTAFSSON, COSTIC ADAM amp; FASHENG ZOU (2022) One new genus and three new species of the Penenirmus-complex (Phthiraptera: Ischnocera) from China, with resurrection of Picophilopterus Ansari, 1947. Zootaxa, 5087: 401426. Zootaxa. 2022 Jun 23;5155(4):600. doi: 10.11646/zootaxa.5155.4.9 | 2022 |
| 3 | Gustafsson DR, Adam C, Zou F. One new genus and three new species of the Penenirmus-complex (Phthiraptera: Ischnocera) from China, with resurrection of Picophilopterus Ansari, 1947. Zootaxa. 2022 Jan 7;5087(3):401-426. doi: 10.11646/zootaxa.5087.3.1 | 2022 |
| 8 | Gustafsson DR, Bush SE. A new subgenus and eight new species of Guimaraesiella Eichler, 1949 (Phthiraptera: Ischnocera: Philopteridae: Brueelia-complex). Zootaxa. 2020 Nov 25;4885(2):zootaxa.4885.2.1. doi: 10.11646/zootaxa.4885.2.1 | 2020 |
| 14 | Gustafsson DR, Bush SE. Chewing lice of the Brueelia-complex (Phthiraptera: Ischnocera) parasitic on members of the Campephagidae (Aves: Passeriformes), with description of a new subgenus and 14 new species. Zootaxa. 2022 Jul 13;5165(1):1-55. doi: 10.11646/zootaxa.5165.1.1 | 2022 |
| 4 | Gustafsson DR, Bush SE. Four new species of Brueelia Kéler, 1936 (Phthiraptera: Ischnocera: Philopteridae) from African songbirds (Passeriformes: Sturnidae and Laniidae). Zootaxa. 2015 Sep 11;4013(4):503-18. doi: 10.11646/zootaxa.4013.4.2 | 2015 |
| 5 | Gustafsson DR, Bush SE. The Genus *Brueelia* (Phthiraptera: Ischnocera: Philopteridae) of North American Jays and Allies (Aves: Passeriformes: Corvidae), with Descriptions of Five New Species. J Parasitol. 2019 Dec;105(6):893-903 | 2019 |
| 3 | Gustafsson DR, Bush SE. Three new species of chewing lice of the genus Emersoniella Tendeiro, 1965 (Insecta: Phthiraptera: Ischnocera: Philopteridae) from Papua New Guinean kingfishers and kookaburras (Aves: Coraciiformes: Alcedinidae). Zootaxa. 2014 May 20;(3796):528-44. doi: 10.11646/zootaxa.3796.3.7 | 2014 |
| 2 | Gustafsson DR, Bush SE. Two new species of Paraphilopterus Mey, 2004 (Phthiraptera: Ischnocera: Philopteridae) from New Guinean bowerbirds (Passeriformes: Ptilonorhynchidae) and satinbirds (Passeriformes: Cnemophilidae). Zootaxa. 2014 Oct 15;3873(2):155-64. doi: 10.11646/zootaxa.3873.2.3 | 2014 |
| 9 | Gustafsson DR, Bush SE. *Brueelia* (Phthiraptera: Ischnocera: Philopteridae) of North American Nine-Primaried Oscines (Aves: Passeriformes: Passerida) with Descriptions of Nine New Species. J Parasitol. 2019 Dec;105(6):858-873 | 2019 |
| 7 | Gustafsson DR, Chu X, Bush SE, Zou F. Seven new species of Resartor Gustafsson et Bush, 2017 (Phthiraptera: Ischnocera: Philopteridae) from Asian 'babblers' (Passeriformes: Leiothrichidae, Paradoxornithidae). Folia Parasitol (Praha). 2018 Dec 20;65:2018.020. doi: 10.14411/fp.2018.020 | 2018 |
| 10 | Gustafsson DR, Chu X, Bush SE, Zou F. Ten new species of Brueelia Kéler, 1936 (Phthiraptera: Ischnocera: Philopteridae) from nuthatches (Aves: Passeriformes: Sittidae), tits and chickadees (Paridae), and goldcrests (Regulidae). Acta Parasitol. 2018 Sep 25;63(3):527-557. doi: 10.1515/ap-2018-0063 | 2018 |
| 12 | Gustafsson DR, Clayton DH, Bush SE. Twelve new species of Guimaraesiella (Phthiraptera: Ischnocera: Philopteridae) from "babblers" (Passeriformes: Leiothrichidae, Pellorneidae, Timaliidae) with a description of a new subgenus and a key to its species. Zootaxa. 2019 Jan 9;4543(4):451-497. doi: 10.11646/zootaxa.4543.4.1 | 2019 |
| 12 | Gustafsson DR, Clayton DH, Bush SE. Twelve new species of Priceiella (Phthiraptera: Ischnocera: Philopteridae) from Old World babblers, with keys to species of two subgenera and checklists of species for the genus. Zootaxa. 2018 Feb 21;4382(3):401-449. doi: 10.11646/zootaxa.4382.3.1 | 2018 |
| 2 | Gustafsson DR, Lei L, Chu X, Zou F, Bush SE. New Genus and Two New Species of Chewing Lice from Southeast Asian Trogons (Aves: Trogoniformes), with a Revised Key to the Philopterus-complex. Acta Parasitol. 2019 Mar;64(1):86-102. doi: 10.2478/s11686-018-00011-x | 2019 |
| 5 | Gustafsson DR, Lei L, Chu X, Zou F. Review of Chinese species of the Oxylipeurus-complex (Phthiraptera: Philopteridae), with descriptions of two new genera and five new species. Zootaxa. 2020 Feb 20;4742(2):zootaxa.4742.2.1. doi: 10.11646/zootaxa.4742.2.1 | 2020 |
| 2 | Gustafsson DR, Lei L, Luo K, Chu X, Zhao X, Zhang Q, Zou F. Chewing lice from high-altitude and migrating birds in Yunnan, China, with descriptions of two new species of Guimaraesiella. Med Vet Entomol. 2019 Sep;33(3):407-419. doi: 10.1111/mve.12378 | 2019 |
| 5 | Gustafsson DR, Malysheva OD, Tolstenkov OO, Bush SE. Five New Species of *Guimaraesiella* (Phthiraptera: Ischnocera) from Broadbills (Aves: Passeriformes: Calyptomenidae: Eurylaimidae). J Parasitol. 2019 Dec;105(6):846-857 | 2019 |
| 4 | Gustafsson DR, Tian C, Ren M, Liu Z, Yu X, Zou F. Four new species of Guimaraesiella (Phthiraptera: Ischnocera: Brueelia-complex) from China. Zootaxa. 2021 Nov 1;5060(3):333-352. doi: 10.11646/zootaxa.5060.3.2 | 2021 |
| 7 | Gustafsson DR, Tian C, Ren M, Liu Z, Yu X, Zou F. NEW SPECIES AND NEW RECORDS OF PRICEIELLA (PHTHIRAPTERA: ISCHNOCERA: BRUEELIA-COMPLEX) FROM SOUTH CHINA. J Parasitol. 2021 Nov 1;107(6):863-877. doi: 10.1645/21-68 | 2021 |
| 5 | Gustafsson DR, Tian C, Zou F. New species of ischnoceran chewing lice (Phthiraptera: Philopteridae) from Chinese birds. Zootaxa. 2021 Jun 21;4990(2):305328. doi: 10.11646/zootaxa.4990.2.6 | 2021 |
| 3 | Gustafsson DR, Tsurumi M, Bush SE. The Chewing Lice (Insecta: Phthiraptera: Ischnocera: Amblycera) of Japanese Pigeons and Doves (Columbiformes), with Descriptions of Three New Species. J Parasitol. 2015 Jun;101(3):304-13. doi: 10.1645/14-683.1 | 2015 |
| 6 | Gustafsson DR, Zou F, Bush SE. Descriptions of six new species of slender-bodied chewing lice of the Resartor-group (Phthiraptera: Ischnocera: Brueelia-complex). Zootaxa. 2022 Mar 1;5104(4):506-530. doi: 10.11646/zootaxa.5104.4.2 | 2022 |
| 1 | Gustafsson DR, Zou F. Descriptions of three congeneric species of chewing lice of the Oxylipeurus-complex (Insecta: Phthiraptera: Philopteridae) from the turkey, Meleagris gallopavo, including a new genus and a new species. Zootaxa. 2020 Jun 19;4801(3):zootaxa.4801.3.4. doi: 10.11646/zootaxa.4801.3.4 | 2020 |
| 3 | Gustafsson DR, Zou F. Species of Reticulipeurus Kéler, 1958 (Phthiraptera, Ischnocera, Oxylipeurus-complex) parasitic on species of Arborophila, with description of a new subgenus and three new species. Zootaxa. 2023 May 12;5284(3):496-520. doi: 10.11646/zootaxa.5284.3.3 | 2023 |
| 2 | Gustafsson DR. New Genus and Two New Species of Chewing Lice (Phthiraptera: Ischnocera) Parasitizing New Guinean *Peltops* (Passeriformes: Artamidae). J Parasitol. 2019 Dec;105(6):840-845 | 2019 |
| 8 | Kolencik S, Sychra O, Papousek I, Kuabara KMD, Valim MP, Literak I. New species and additional data on the chewing louse genus Myrsidea (Phthiraptera: Menoponidae) from wild Neotropical Passeriformes (Aves). Zootaxa. 2018 May 10;4418(5):401-431. doi: 10.11646/zootaxa.4418.5.1 | 2018 |
| 7 | Kounek F, Sychra O, Capek M, Literak I. Chewing lice of genus Myrsidea (Phthiraptera: Menoponidae) from Turdidae (Passeriformes) of Costa Rica, with descriptions of seven new species. Zootaxa. 2013;3620:201-22. doi: 10.11646/zootaxa.3620.2.1 | 2013 |
| 4 | Lei L, Chu X, Dik B, Zou F, Wang H, Gustafsson DR. Four new species of Myrsidea (Phthiraptera: Amblycera: Menoponidae) from Chinese babblers (Passeriformes: Leiothrichidae, Paradoxornithidae, Timaliidae). Zootaxa. 2020 Nov 12;4878(1):zootaxa.4878.1.4. doi: 10.11646/zootaxa.4878.1.4 | 2020 |
| 2 | Najer T, Gustafsson DR, Sychra O. Two new species of Philopteroides (Phthiraptera: Ischnocera: Philopteridae) of the beckeri species-group, from New Guinean painted berrypeckers (Aves: Passeriformes: Paramythiidae). Zootaxa. 2016 Jul 22;4139(4):527-41. doi: 10.11646/zootaxa.4139.4.5 | 2016 |
| 2 | Najer T, Sychra O, Kounek F, Papousek I, Hung NM. Chewing lice (Phthiraptera: Amblycera and Ischnocera) from wild birds in southern Vietnam, with descriptions of two new species. Zootaxa. 2014 Jan 24;3755:419-33. doi: 10.11646/zootaxa.3755.5.2 | 2014 |
| 3 | Najer T, Sychra O, Literák I, Procházka P, Capek M, Koubek P. Chewing lice (Phthiraptera) from wild birds in Senegal, with descriptions of three new species of the genera Brueelia and Philopteroides. Acta Parasitol. 2012 Mar;57(1):90-8. doi: 10.2478/s11686-012-0005-x | 2012 |
| 1 | Naz S, Sychra O, Rizvi SA. New records and a new species of chewing lice (Phthiraptera, Amblycera, Ischnocera) found on Columbidae (Columbiformes) in Pakistan. Zookeys. 2012;(174):79-93. doi: 10.3897/zookeys.174.2717 | 2012 |
| 2 | Román-P C, García Aldrete AN, Obando RG. Elaphopsocoides, a new genus of Psocidae (Psocodea: ´Psocoptera´) from Valle del Cauca, Colombia. Zootaxa. 2014 Oct 14;3873(1):93-100. doi: 10.11646/zootaxa.3873.1.8 | 2014 |
| 1 | Shrestha P, Dik B, Maharjan M. Chewing lice from the white-rumped vulture in Nepal, with description of a new species of Aegypoecus. Zootaxa. 2019 Nov 4;4691(5):zootaxa.4691.5.4. doi: 10.11646/zootaxa.4691.5.4 | 2019 |
| 3 | Sychra O, Kolencik S, Palma RL. Three new species of Myrsidea (Phthiraptera: Menoponidae) from New Zealand passerines (Aves: Passeriformes). Zootaxa. 2016 Jun 20;4126(3):397-410. doi: 10.11646/zootaxa.4126.3.5 | 2016 |
| 1 | Sychra O, Kounek F, Capek M, Literak I. Myrsidea povedai (Phthiraptera: Menoponidae), a new species of chewing louse from Phainoptila melanoxantha (Passeriformes: Bombycillidae). J Parasitol. 2011 Aug;97(4):593-5. doi: 10.1645/GE-2715.1 | 2011 |
| 1 | Sychra O, Najer T, Kounek F, Capek M, Literak I. Chewing lice (Phthiraptera) on manakins (Passeriformes: Pipridae) from Costa Rica, with description of a new species of the genus Tyranniphilopterus (Phthiraptera: Philopteridae). Parasitol Res. 2010 Mar;106(4):925-31. doi: 10.1007/s00436-010-1768-3 | 2010 |
| 1 | Sychra O, Palma RL. A new species of Myrsidea (Insecta: Phthiraptera: Menoponidae) from Chile. Zootaxa. 2021 Aug 6;5016(3):441-447. doi: 10.11646/zootaxa.5016.3.9 | 2021 |
| 1 | Sychra O, Palma RL. Erratum: OLDRICH SYCHRA amp; RICARDO L. PALMA (2021) A new species of Myrsidea (Insecta: Phthiraptera: Menoponidae) from Chile. Zootaxa, 5016: 441447. Zootaxa. 2022 Jan 10;5087(4):600. doi: 10.11646/zootaxa.5087.4.9 | 2022 |
| 1 | Valan M, Sychra O, Literak I. Chewing lice of genus Ricinus (Phthiraptera, Ricinidae) deposited at the Zoological Institute of the Russian Academy of Sciences, Saint Petersburg, Russia, with description of a new species. Parasite. 2016;23:7. doi: 10.1051/parasite/2016007 | 2016 |
| 1 | Valim MP, Cicchino AC. A remarkable new genus and a new species of chewing louse (Phthiraptera, Ischnocera, Philopteridae) from Brazil. Zookeys. 2015 Dec 1;(541):57-70. doi: 10.3897/zookeys.541.6022 | 2015 |
| 6 | Valim MP, Cicchino AC. Six new species of Myrsidea Waterston, 1915 (Phthiraptera: Menoponidae) from New World jays of the genus Cyanocorax Boie (Passeriformes: Corvidae), with notes on the chorionic structure of eggs. Syst Parasitol. 2015 Feb;90(2):191-211. doi: 10.1007/s11230-014-9543-y | 2015 |
| 5 | Valim MP, Kuabara KM. The feather louse genus Mulcticola Clay et Meinertzhagen, 1938 (Phthiraptera: Philopteridae) from Brazil, with descriptions of five new species and catalogue for species described in the genus. Folia Parasitol (Praha). 2015 Jul 22;62:2015.036. doi: 10.14411/fp.2015.036 | 2015 |
| 2 | Valim MP, Palma RL. A new genus and two new species of feather lice (Phthiraptera: Ischnocera: Philopteridae) from New Zealand endemic passerines (Aves: Passeriformes). Zootaxa. 2015 Mar 9;3926(4):480-98. doi: 10.11646/zootaxa.3926.4.2 | 2015 |
| 3 | Valim MP, Palma RL. Three new species of the genus Philopteroides Mey, 2004 (Phthiraptera, Ischnocera, Philopteridae) from New Zealand. Zookeys. 2013 May 10;(297):71-89. doi: 10.3897/zookeys.297.5118 | 2013 |
| 1 | Valim MP, Silveira LF. A new species and five new records of chewing lice (Insecta: Phthiraptera: Ischnocera) from an isolated population of the solitary tinamou Tinamus solitarius (Aves: Tinamiformes). Zootaxa. 2014 Jul 16;3838(1):127-42. doi: 10.11646/zootaxa.3838.1.8 | 2014 |
| 10 | Valim MP, Weckstein JD. A drop in the bucket of the megadiverse chewing louse genus Myrsidea (Phthiraptera, Amblycera, Menoponidae): ten new species from Amazonian Brazil. Folia Parasitol (Praha). 2013 Nov;60(5):377-400. doi: 10.14411/fp.2013.040 | 2013 |
| 1 | Valim MP, Weckstein JD. A new genus and species of Philopteridae (Phthiraptera: Ischnocera) from the trumpeters (Aves: Gruiformes: Psophiidae). J Parasitol. 2012 Aug;98(4):728-34. doi: 10.1645/GE-3055.1 | 2012 |
| 2 | Valim MP, Weckstein JD. Two new species of Brueelia Kéler, 1936 (Ischnocera, Philopteridae) parasitic on Neotropical trogons (Aves, Trogoniformes). Zookeys. 2011;(128):1-13. doi: 10.3897/zookeys.128.1583 | 2011 |
| 2 | Valim MP, Weckstein JD. Two new species of Cotingacola Carriker, Phthiraptera: Ischnocera: Philopteridae) from Amazonian Brazil, with comments on host-specificity. Syst Parasitol. 2012 Mar;81(3):159-67. doi: 10.1007/s11230-011-9334-7 | 2012 |

**Supplementary Fig. 1**

**
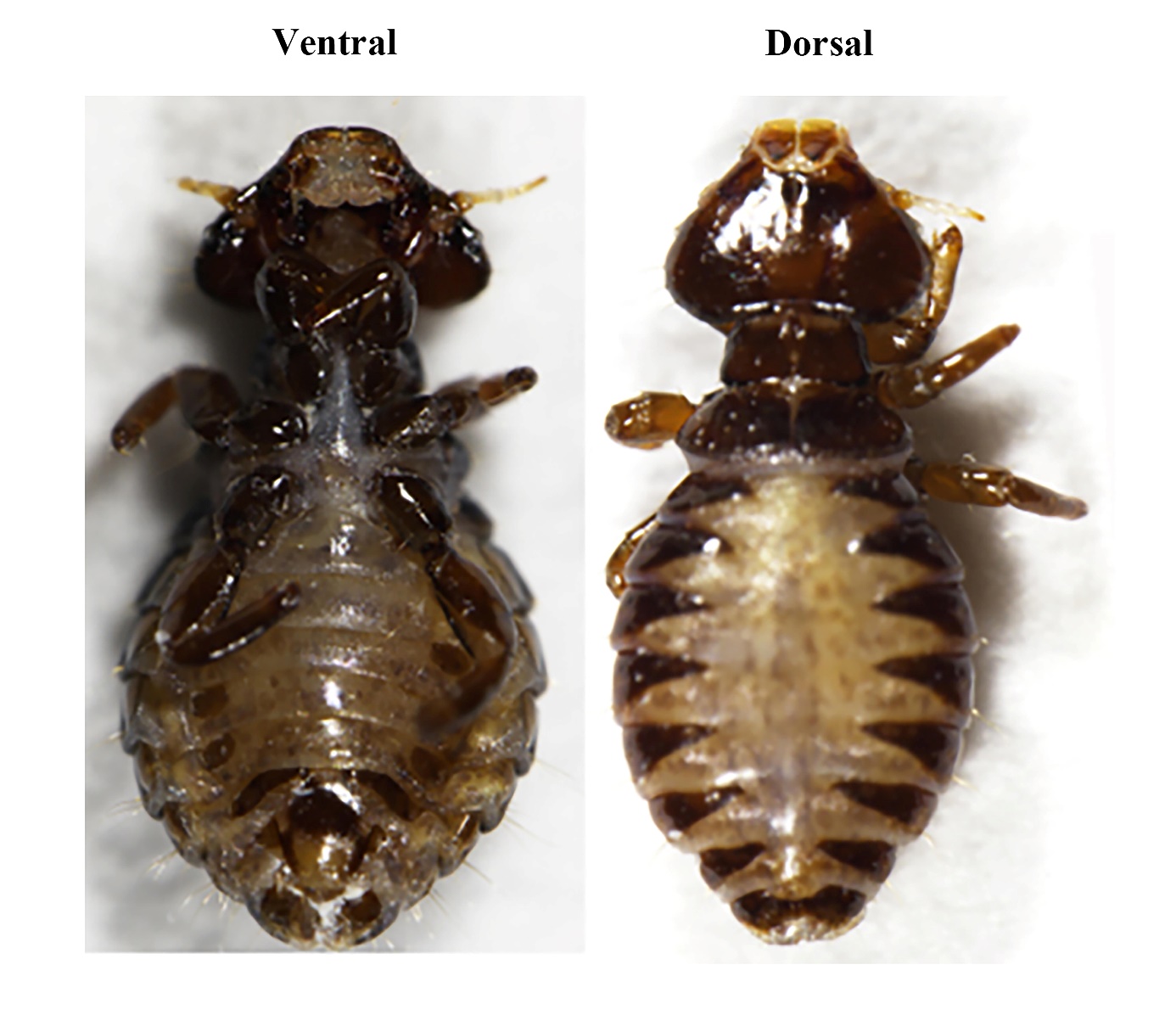
**

**Supplementary Fig. 2**

**
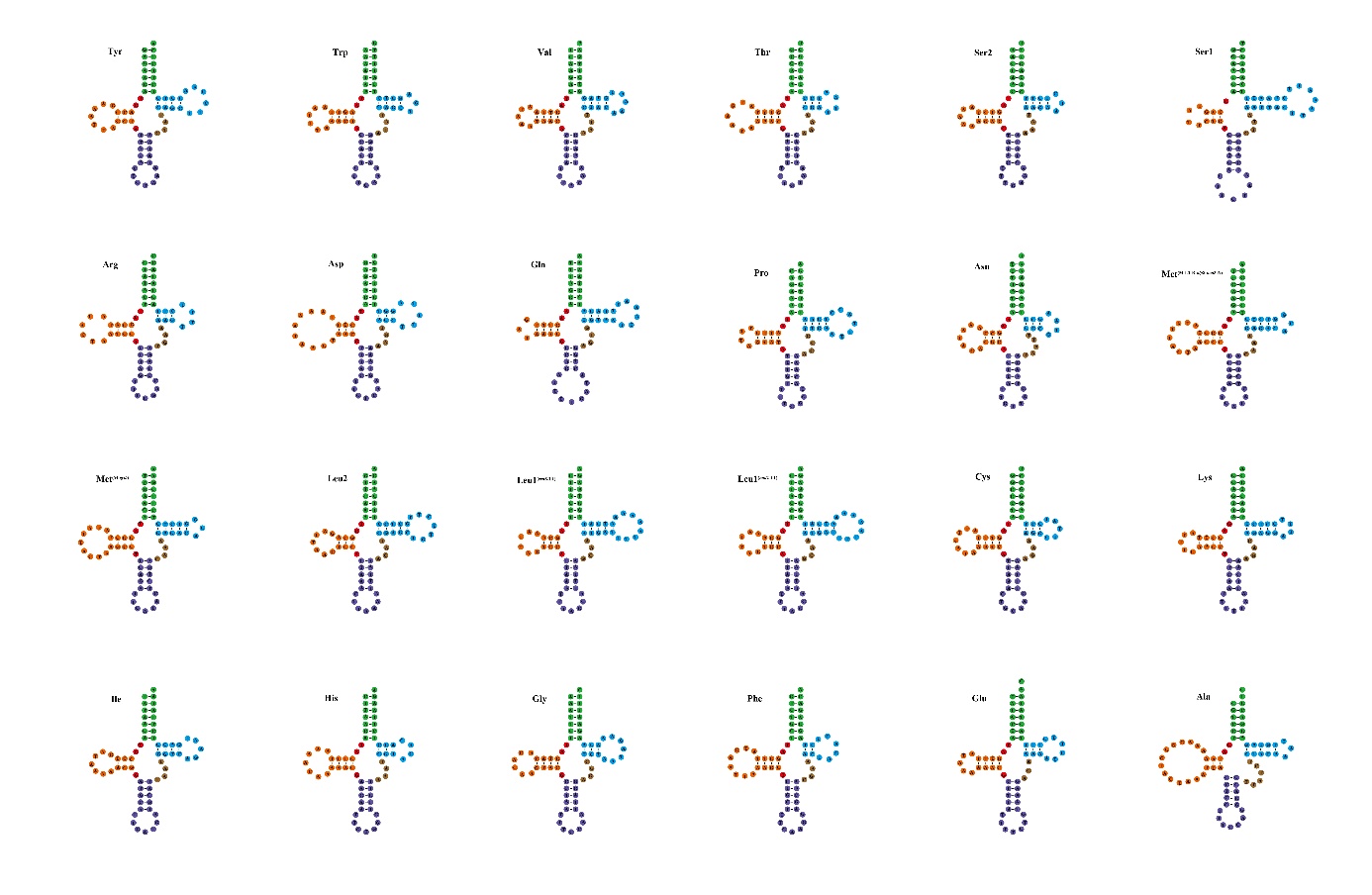
**
